# Probing and imaging phospholipid dynamics in live cells

**DOI:** 10.1093/lifemeta/loae014

**Published:** 2024-04-13

**Authors:** Zhongsheng Wu, Yongtao Du, Tom Kirchhausen, Kangmin He

**Affiliations:** State Key Laboratory of Molecular Developmental Biology, Institute of Genetics and Developmental Biology, Chinese Academy of Sciences, Beijing 100101, China; University of Chinese Academy of Sciences, Beijing 100049, China; State Key Laboratory of Molecular Developmental Biology, Institute of Genetics and Developmental Biology, Chinese Academy of Sciences, Beijing 100101, China; University of Chinese Academy of Sciences, Beijing 100049, China; Department of Cell Biology, Harvard Medical School, Boston, MA 02115, United States; Program in Cellular and Molecular Medicine, Boston Children’s Hospital, Boston, MA 02115, United States; Department of Pediatrics, Harvard Medical School, Boston, MA 02115, United States; State Key Laboratory of Molecular Developmental Biology, Institute of Genetics and Developmental Biology, Chinese Academy of Sciences, Beijing 100101, China; University of Chinese Academy of Sciences, Beijing 100049, China

**Keywords:** phospholipid, biosensor, lipid-binding domain, live-cell imaging

## Abstract

Distinct phospholipid species display specific distribution patterns across cellular membranes, which are important for their structural and signaling roles and for preserving the integrity and functionality of the plasma membrane and organelles. Recent advancements in lipid biosensor technology and imaging modalities now allow for direct observation of phospholipid distribution, trafficking, and dynamics in living cells. These innovations have markedly advanced our understanding of phospholipid function and regulation at both cellular and subcellular levels. Herein, we summarize the latest developments in phospholipid biosensor design and application, emphasizing the contribution of cutting-edge imaging techniques to elucidating phospholipid dynamics and distribution with unparalleled spatiotemporal precision.

## Introduction

Lipids are not only the fundamental building blocks of cellular membranes and energy storage reservoirs, but they can also function as second messengers in signaling transduction, regulate membrane traffic, and define organelle identity [1]. Mammalian cells contain more than a thousand different lipid species. Most phospholipids are synthesized in the endoplasmic reticulum (ER) and subsequently distributed across various organelle membranes [2, 3]. Recent rapid advancements in mass spectrometry- and microscopy-based techniques have unveiled that different lipid species have distinct subcellular distribution and kinetics, and different organelles have characteristic lipid compositions.

Glycerophospholipids, including phosphatidylcholine (PC), phosphatidylethanolamine (PE), phosphatidylserine (PS), and phosphatidylinositol (PI), are the main phospholipids and major building constituents of cellular membranes [3]. Reversible phosphorylation of PI at specific positions (-3, -4, or -5) of the myo-inositol head ring gives rise to seven distinct but interconvertible phosphoinositide species in eukaryotic cells, namely PtdIns3P, PtdIns4P, PtdIns5P, PtdIns(3,4)P_2_, PtdIns(3,5)P_2_, PtdIns(4,5)P_2_, and PtdIns(3,4,5)P_3_ [4]. Although these phosphoinositides constitute less than 1% of total phospholipids, they play vital roles in cell signaling transduction and membrane trafficking [4–8]. Each phospholipid species exhibits a unique subcellular distribution and dynamics, which are essential for the function and identity of various organelles [4, 6]. PS is the most abundant anionic phospholipid in cellular membranes and plays a crucial role in maintaining plasma membrane homeostasis and facilitating protein targeting to the plasma membrane and the endocytic pathway [9]. Under circumstances such as apoptosis, mast cell degranulation, and platelet activation, the asymmetric distribution of PS is disrupted to trigger phagocytosis and accelerate blood clotting [10–12]. Phosphatidic acid (PA), a less abundant anionic phospholipid, is primarily found in the plasma membrane, ER, mitochondria, and nuclear membrane [10, 13]. PA possesses distinctive biophysical properties (including charge and shape) and acts as a signaling molecule and a building block for the synthesis of other lipids [14–16].

The generation and turnover of lipids are tightly regulated by lipid kinases, phosphatases, and phospholipases. Mutations of genes encoding lipid metabolism enzymes can lead to aberrant lipid generation, turnover, or distribution, which have been implicated in various human diseases, including cancer, cardiovascular diseases, neurological disorders, diabetes, and developmental disorders [17–19]. For instance, PtdIns(4,5)P_2_ is a phospholipid located predominantly at the inner leaflet of the plasma membrane. A minor population of PtdIns(4,5)P_2_ has also been found at the outer leaflet of the plasma membrane, Golgi, endosomes, ER, and nucleus [20, 21]. Genetic mutations in the inositol-5-phosphatase OCRL1 (oculocerebrorenal syndrome of Lowe) resulted in abnormal accumulation of PtdIns(4,5)P_2_ in endolysosome pathways, causing delayed endocytic recycling of receptors and impaired lysosomal–autophagic dynamics and functions [22–24]. Dysregulation of PtdIns(4,5)P_2_ may contribute to the proximal tubulopathy in Lowe syndrome and Dent-2 disease, both of which are caused by OCRL1 mutations [23, 24]. PtdIns(3,4,5)P_3_ is an extremely low-abundance phospholipid primarily located at the cytoplasmic leaflet of the plasma membrane. Dysregulation in PtdIns(3,4,5)P_3_ generation and turnover has been associated with various human diseases, including cancer, diabetes, cardiovascular diseases, and inflammation [25–28]. Thus, gaining a comprehensive understanding of the subcellular distribution, trafficking, and turnover of phospholipids in live cells is important for elucidating their function in regulating various cellular activities, as well as their roles in human diseases.

Over the years, researchers have developed a variety of molecular probes for lipid detection in cells [29, 30]. These molecular probes, assisted by advanced imaging techniques, have significantly enhanced our understanding of the subcellular distribution, kinetics, and metabolism of various phospholipid species in cells. Here, we review current strategies used in the design of phospholipid biosensors compatible with live-cell imaging and discuss imaging techniques utilized to track and analyze the dynamic behavior of phospholipids in live cells.

## Genetically encoded lipid-binding domains

As has been summarized in several excellent reviews, the molecular probes for phospholipid detection in fixed and live cells include fluorophore-conjugated lipids, antibodies, toxin domains, and genetically encoded lipid-binding protein domains [29, 30]. Among these, genetically encoded lipid-binding domains are the most widely utilized for lipid detection (sometimes they are the only available tools) in live cells. They serve as the fundamental building blocks for designing lipid sensors using various strategies (see Section “Phospholipid biosensor design”). Here, we provide a summary of existing lipid-binding domains for the signaling phosphoinositides, PS, and PA.

The FYVE (Fab1, YOTB, Vac1, and EEA1) domain of hepatocyte growth factor-regulated tyrosine kinase substrate (Hrs) or early endosomal antigen 1 (EEA1), which specifically binds to PtdIns3P, is the most commonly used lipid-binding domain for PtdIns3P [31–33]. The PtdIns4P binding domain of SidM/DrrA (P4M) or SidC (P4C) exhibits high affinity and specificity for PtdIns4P and has been used to create biosensors for detecting PtdIns4P in live cells [34–37]. The pleckstrin homology (PH) domain of phospholipase Cδ1 (PLCδ1) is the most widely used protein domain for PtdIns(4,5)P_2_ detection and has also demonstrated an affinity for inositol 1,4,5-trisphosphate (IP3) [38, 39]. Another probe for monitoring PtdIns(4,5)P_2_, particularly following phospholipase C activation, is the C-terminal domain of Tubby (Tubby_c_) [39, 40]. It should be noted that this domain also binds to PtdIns(3,4,5)P_3_ and PtdIns(3,4)P_2_ [41]. The monomeric or tandem forms of the entire C-terminal PH domain or the triple tandem of the isolated C-terminal PH domain of tandem PH-domain-containing protein 1 (TAPP1) have been employed to detect PtdIns(3,4)P_2_ in live cells [42–45]. The widely used PtdIns(3,4,5)P_3_-binding domains include the PH domains of Bruton’s tyrosine kinase (Btk), protein kinase B (Akt) (also binds to PtdIns(3,4)P_2_), general receptor for phosphoinositides 1 (GRP1) (also binds to the ADP-ribosylation factor (Arf)/Arf-like (Arl)), and the tandem PH domain of Arf nucleotide-binding site opener ARNO^2G-I303E^ [29, 44, 46–49]. The triple repeat of the plant homeodomain (PHD) domain of inhibitor of growth 2 (ING2) shows specific binding to PtdIns5P and has been used to detect PtdIns5P in cells [50, 51]. The tandem repeat of the phosphoinositide-interacting domain (ML1N) of transient receptor potential Mucolipin 1 (TRPML1) shows specific binding to PtdIns(3,5)P_2_ *in vitro* and has been used to detect PtdIns(3,5)P_2_ in cells, although its selectivity in cells requires further evaluation [52, 53]. The *Dictyostelium* protein Senexin A (SnxA) exhibits high specificity in binding to PI(3,5)P_2_, and SnxA or the tandem repeat of its lipid-binding Phox homology (PX)-domain could serve as new reporters for PI(3,5)P_2_ [54]. The C-terminal Src-homology 2 (SH2) domains (cSH2) of the regulatory p85α subunit of class I phosphoinositide 3-kinase (PI3K) were found to have a high affinity for PI(3,5)P_2_ and have been utilized in developing PI(3,5)P_2_-specific ratiometric fluorescence sensor [55]. Two recent studies have developed PI-specific sensors either through chemically induced dimerization of a split PI-specific phospholipase C (PI-PLC) from *Listeria monocytogenes* or by reengineering the PI-PLC from *Bacillus cereus* [56, 57]. The C2 domain of lactadherin (Lact-C2) and the tandem repeat of the PH domain of evectin-2 have been widely employed to visualize the subcellular distribution and dynamics of PS in live cells [58–60]. The monomeric or tandem repeat of the PA-binding domain of Spo20p, tagged with a nuclear export sequence and a fluorescent protein at the N-terminus, has been used as a specific sensor to detect PA [61].

As extensively discussed in multiple reviews, there are potential caveats associated with the overexpression of these chimeras [30, 39, 62–64]. For example, the expression of lipid-binding domains at a high level may sequester lipids or interfere with the binding of endogenous effector proteins to target lipids. Thus, several practical considerations must be taken into account for the successful application of these sensors, including controlling sensor expression levels, utilizing mutant sensors deficient in lipid binding, selecting more sensitive and appropriate imaging tools, and interpreting the imaging data with caution [30, 39, 62–64].

## Phospholipid biosensor design

**Figure 1:**
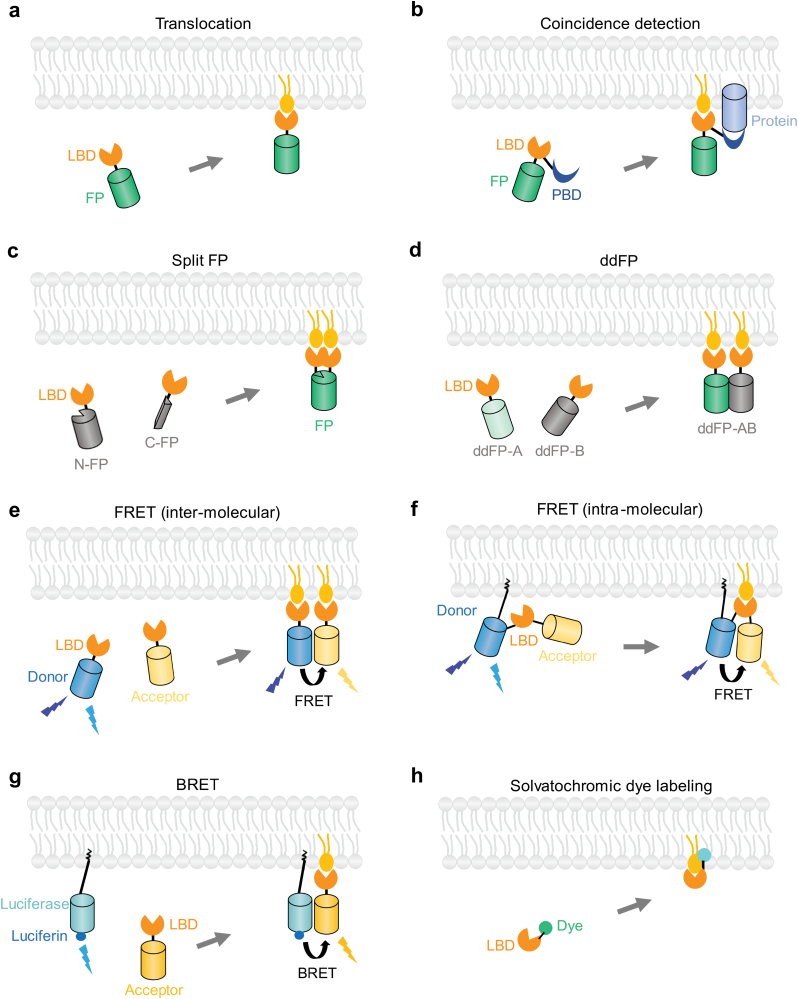
Schematic overview of phospholipid biosensors based on lipid-binding domains currently used for detecting phospholipids in live cells. The design principles are shown for the biosensors based on protein domain translocation (a), coincidence detection (b), split fluorescent protein (FP) (c), ddFPs (d), inter-molecular or intra-molecular FRET (e and f), BRET (g), and chemical labeling of the lipid-binding protein domain with solvatochromic fluorophores (h). See texts for details. LBD, lipid-binding domain; PBD, protein-binding domain; N-FP: N-terminal part of a fluorescent protein; C-FP: C-terminal part of a fluorescent protein.

Here, we focus on the various strategies employed to design phospholipid biosensors, which utilize lipid-binding protein domains (Fig. 1; Table 1). We explore the design principles, characteristics, and applications of these biosensors.

**Table 1.** Summary of phospholipid biosensors designed using different strategies.

Design strategy	Phospholipid	Biosensor	Advantage	Limitation	Note	Reference
Translocation (illustrated with EGFP tagging)	PtdIns3P	EGFP-2xFYVE(Hrs) EGFP-FYVE(EEA1)	Easy to construct and well characterized. Do not require sophisticated imaging tools. Different fluorescent tags enable multi-color imaging	High levels of sensor expression may lead to inhibitory effects, such as lipid sequestration, competition with endogenous proteins, and membrane deformation		[31–33]
	PtdIns4P	EGFP-P4M(SidM) EGFP-P4C(SidC)			SidM is also called DrrA	[34–37]
	PtdIns(4,5)P_2_	EGFP-PH(PLCδ1) EGFP-Tubby_c_			PH(PLCδ1) also binds IP3; Tubby_c_ also binds PtdIns(3,4)P_2_ and PtdIns(3,4,5)P_3_	[38–40]
	PtdIns(3,4)P_2_	NES-EGFP-3xcPH(TAPP1) EGFP-2xPH(TAPP1)			NES: nuclear export sequence	[42–45]
	PtdIns(3,4,5)P_3_	EGFP-PH(Btk) NES-EGFP-2xPH(ARNO^2G-I303E^) PH(Akt)-EGFP EGFP-PH(GRP1)			PH(Akt) also binds PtdIns(3,4)P_2_; PH(GRP1) also binds Arf/Arl	[29, 44, 46–49]
	PtdIns5P	EGFP-3xPHD(ING2)			3xPHD(ING2) also binds PtdIns3P	[50, 51]
	PtdIns(3,5)P_2_	EGFP-2xPX(SnxA) EGFP-SnxA EGFP-2xML1N(TRPML1)			2xML1N(TRPML1) may have nonspecific binding in mammalian cells	[52–54]
	PI	EGFP-*Bc*PI-PLC^H82A^ EGFP-*Bc*PI-PLC^ANH^			Catalytically dead PI-PLC from *Bacillus cereus*	[56]
	PS	EGFP-Lact-C2 EGFP-2xPH(evectin-2)				[58–60]
	PA	EGFP-NES-PABD(Spo20)				[61]
Coincidence detection	PtdIns3P	EGFP-2xFYVE(Hrs)-Aux1	Enables organelle-specific detection. Increased affinity allows lower sensor expression	Detection of the presence but not concentration of lipids	Auxilin1-based lipid biosensors are specific for clathrin-coated structures	[65]
	PtdIns4P	EGFP-P4M(DrrA)-Aux1				
	PtdIns(3,4)P_2_	EGFP-2xPH(TAPP1)-Aux1				
	PtdIns(4,5)P_2_	EGFP-PH(PLCδ1)-Aux1				
Split protein domains	PtdIns(3,4,5)P_3_	VenusC-PH(GRP1) with VenusN-PH(GRP1)	Low background signal	May exhibit false-positive signals due to nonspecific reassembly		[66]
	PI	PI-PLC^C100^-FRB with FKBP-PI-PLC^N187^ and DAG sensor NES-EGFP-C1ab(PKD1)	Enables organelle-specific detection	Indirect evaluation of PI levels by measuring DAG production	PI-PLC from *Listeria monocytogenes*	[57]
ddFP	PtdIns(4,5)P_2_	RA-PH(PLCδ1) with B-PH(PLCδ1) and GA	Low background. Enables organelle-specific detection	Low fluorescence. Not suitable for phospholipids with low abundance	GA and RA: green and red fluorescent versions of ddFP. B: copy B of ddFP	[67]
FRET	PtdIns(4,5)P_2_ (inter-molecular)	PH(PLCδ1)-CFP with PH(PLCδ1)-YFP	Enables organelle-specific detection Suitable for cell populations and membrane protrusions of single cells	Requirements for specific hardware and data analysis	MLS: membrane localization sequence	[68]
	PtdIns(3,4,5)P_3_ (intra-molecular)	CFP-PH(GRP1)-YFP-MLS				[69]
BRET	PtdIns4P	PM-Venus-T2A-Luciferase-2xP4M(SidM)	No excitation light required. Suitable for cell populations or high-throughput imaging	Weak signal from individual cells	PM: plasma membrane-targeting sequence	[70–73]
	PtdIns(4,5)P_2_	PM-Venus-T2A-PH(PLCδ1)-Luciferase				
	PtdIns(3,4,5)P_3_	PM-Venus-T2A-PH(Btk)-Luciferase PM-Venus-T2A-PH(GRP1)-Luciferase				
	PtdIns(3,4)P_2_	PM-Venus-T2A-2xPH(TAPP1)-Luciferase				
	PS	PM-Venus-T2A-Luciferase-Lact-C2				
Chemical labeling of lipid-binding domain	PtdIns(3,4)P_2_	NR3-eTapp1-cPH	Quantitative measurement of lipid distribution and concentration. Quantitative imaging of lipids in both leaflets of the plasma membrane	Requirement for subtle protein engineering. Quantification accuracy relies on the sensitivity and specificity of the lipid-binding domain	NR3, DAN, and WCB are environment-sensitive solvatochromic fluorophores	[42, 55, 74–76]
	PtdIns(4,5)P_2_	NR3-eENTH DAN-eENTH WCB-eENTH				
	PtdIns(3,4,5)P_3_	NR3-eMyoX-PH WCR-eMyoX-tPH				
	PtdIns(3,5)P_2_	WCB-ep85α-cSH2				
	PS	DAN-eLact-C2 NR3-eLact-C2				

### Phospholipid biosensors based on translocation

The simplest lipid sensors are based on the translocation of fluorescent sensors from the cytoplasm to the plasma membrane or organelles upon stimulation. These sensors are created by fusing fluorescent proteins or dyes with lipid-binding domains; visualization is then carried out using fluorescence microscopy. The sensor is recruited to the membrane upon local generation of the target lipid, whereas depletion of the lipid results in sensor dissociation from the membrane (Fig. 1a). This allows the determination of relative lipid concentration or turnover at specific cellular membranes by measuring the relative increase or decrease of fluorescence intensity, or by calculating the fluorescence ratio between cytosol and the targeted membrane [46].

Early studies demonstrated that the PH domain of PLCδ1 exhibits binding specificity for PtdIns(4,5)P_2_-containing lipid bilayers or vesicles [77, 78]. A significant breakthrough came with the fusion of the PH domain of PLCδ1 to the N- or C- terminus of green fluorescent protein (GFP), enabling monitoring of PtdIns(4,5)P_2_ distribution and dynamics in live cells [38, 79]. The fusion protein was rapidly recruited from the cytoplasm to the PtdIns(4,5)P_2_-enriched plasma membrane, while constructs with point mutations in the lipid-binding pocket failed to be recruited. Hydrolysis of PtdIns(4,5)P_2_ by endogenous PLC activation or acute recruitment of an inositol 5-phosphatase domain to the plasma membrane resulted in the rapid dissociation of the sensor from the plasma membrane [38, 79, 80]. This design, involving the fusion of lipid-binding domains with fluorescent proteins, was subsequently adapted to generate biosensors for various other lipids, including PtdIns(3,4,5)P_3_, PtdIns4P, and PS [34, 47, 58].

To enhance the avidity of translocation-based sensors, tandem dimers or even trimers of lipid-binding domains have been employed [34, 44, 81]. However, caution must be exercised as these tandem lipid-binding domains can also exert inhibitory effects on cells, especially at high expression levels. It is always recommended to maintain low expression levels of the sensors and utilize imaging tools with higher sensitivity. Another approach to increase detection sensitivity is to utilize brighter fluorescent proteins or self-labeling proteins. mNeonGreen, a monomeric green or yellow fluorescent protein, is approximately three times brighter than the commonly used monomeric enhanced GFP (mEGFP) [82]. StayGold and its monomeric StayGold variants, including mStayGold, StayGold-E138D, and mBaoJin, are recently developed bright GFPs with exceptional photostability [83–87]. Bright monomeric red fluorescent proteins, such as mScarlet and its variant mScarlet-I, and the recently engineered FusionRed-MQV and mScarlet3, are several times brighter than mCherry in live cells [88–90]. Fusing these significantly brighter fluorescent proteins with lipid-binding domains enables sensor expression at lower levels and the use of lower laser power for live-cell imaging. Genetically encoded self-labeling enzyme tags, such as HaloTag and SNAP-tag, can fuse with lipid-binding domains and then react with their selective functionalized substrates [91, 92]. The substrates, tagged with different membrane-permeable organic fluorophores, provide versatility in labeling lipids with different colors without modifying the fusion proteins and enable simultaneous labeling of two or three different lipid species. Janelia Fluor dyes with excellent brightness, photostability, and cell permeability are examples of ideal fluorophores for detection at both the ensemble and single-molecule levels [93, 94].

### Phospholipid biosensors based on coincidence detection

The lipid-binding domains of most lipid-binding proteins exhibit relatively weak binding to phospholipids [95, 96]. Specific subcellular recruitment of lipid-binding proteins requires the simultaneous binding of the protein to both the lipid and other factors, such as proteins, lipids, or geometric cues [6, 95, 97]. This mechanism of coincidence detection is commonly observed in phospholipid signaling.

**Figure 2:**
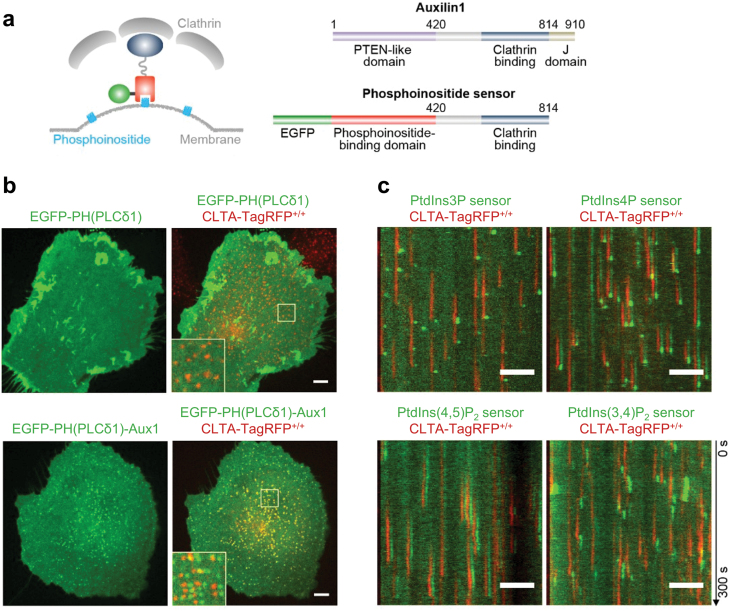
Coincidence detection sensors selectively report the phosphoinositide composition of endocytic clathrin-coated structures. (a) Diagram illustrating the design of coincidence detecting phosphoinositide sensors specific to clathrin-coated structures. (b) Localization of a general PtdIns(4,5)P_2_ sensor (EGFP–PH(PLCδ1)) and an Auxilin1-based PtdIns(4,5)P_2_ sensor (EGFP–PH(PLCδ1)-Aux1) in genome-edited SUM159 cells expressing clathrin light chain A (CLTA)-TagRFP), imaged using spinning-disk confocal microscopy. The EGFP channel in the enlarged regions was laterally shifted by six pixels. (c) Kymographs show the recruitment of the indicated Auxilin1-based phosphoinositide sensors (EGFP-tagged) in CLTA–TagRFP^+/+^ cells imaged by TIRF microscopy. The EGFP channel in the kymographs was laterally shifted by six pixels. Scale bars, 5 μm. Images reproduced with permission from [65].

The majority of lipid biosensors currently in use are based solely on the lipid-binding domain *per se*. However, they are unable to detect the presence or dynamics of low-abundance anionic phospholipids, such as phosphoinositides and PA, within small and dynamic cellular structures like clathrin-coated pits. By taking advantage of the coincidence detection strategy, lipid biosensors can be created by fusing lipid-binding domains with organelle-specific targeting sequences, enabling their high affinity binding to specific subcellular membranes at low biosensor concentration (Fig. 1b). This strategy has been employed to design phosphoinositide sensors specific to clathrin-associated structures [65]. Through the fusion of a phosphoinositide-binding domain of known specificity with the clathrin-binding domain from Auxilin1, it was possible to generate sensors to detect phosphoinositides (PtdIns3P, PtdIns4P, PtdIns(3,4)P_2_, and PtdIns(4,5)P_2_) [65]. These coincidence detection-based sensors exhibit specificity toward their targeted lipids, as point mutations in the lipid-binding domains effectively abolished their recruitment to clathrin-coated structures. Live-cell imaging using total internal reflection fluorescence (TIRF) microscopy with single-molecule sensitivity revealed distinct recruitment dynamics for each sensor and a programmed series of phosphoinositide conversions during the assembly–disassembly cycle of clathrin-mediated endocytosis [65, 98] (Fig. 2). The coincidence detection strategy can be applied in the design of lipid biosensors specific to other subcellular structures or organelles, such as endosomes. Careful evaluation of the specificity and selectivity of the organelle-targeting domains is crucial to achieve specific targeting while avoiding potential inhibitory effects on the organelle.

### Phospholipid biosensors based on split protein domains

Phospholipid sensors can also be designed by splitting either the fluorescent tag or the lipid-binding domain [57, 66]. In the case of phospholipids with very low abundance, such as PtdIns(3,4,5)P_3_ in resting cells, the translocation or fluctuation of lipid sensors at the cellular membrane is minimal. The high fluorescence background arising from unbound sensors compromises reliable measurement. For instance, the EGFP-tagged PH domain of GRP1 was found to be distributed throughout the boutons of transgenic flies, making it challenging to assess PtdIns(3,4,5)P_3_ localization accurately at synapses [66]. To overcome this limitation, a PtdIns(3,4,5)P_3_ probe set based on split Venus was developed by fusing the N- and C-terminal ends of Venus separately with the PH domain of GRP1 [66] (Fig. 1c). Recovery of Venus fluorescence occurred when the two split fragments were bound to locally concentrated PtdIns(3,4,5)P_3_. Through super-resolution imaging, this sensor enabled the visualization of discrete PtdIns(3,4,5)P_3_ foci at *Drosophila* larval neuromuscular synapses [66]. A similar strategy has been applied with lipid sensors based on Förster resonance energy transfer (FRET) or bioluminescence resonance energy transfer (BRET), as discussed below. Thus, the application of split fluorescent proteins can help reduce the background signal of fluorescent translocation-based biosensors. Furthermore, the two parts of the split fluorescent protein can be fused separately to a lipid-binding domain and an organelle-targeting domain to achieve organelle-specific lipid detection. However, it should be noted that the irreversible complementation and nonspecific self-assembly of split fragments (especially at high expression levels) may lead to false-positive lipid detection [99]. Careful control experiments are necessary to interpret the results. The development of new sets of split fluorescent proteins [100–102] or split self-labeling tags [103] is expected to further enhance the specificity and versatility of lipid sensors based on split fluorescent domains.

Enzymatic lipid-binding domains or proteins, such as the bacterial PI-PLC, are able to bind to and subsequently catalyze the conversion or degradation of the targeted lipid [104]. Consequently, a direct fusion of fluorescent proteins with bacterial PI-PLC to measure PI is not feasible. To eliminate the catalytic activity of the *Bacillus cereus* PI-PLC, rational mutagenesis based on protein structures was employed, resulting in the generation of the PI sensor [56]. Another strategy is to create an acutely activatable PI-PLC by fusing the N-terminal domain of *Listeria monocytogenes* PI-PLC with the FKBP (FK506-binding protein) domain and the C-terminal domain of PI-PLC with the FRB (FKBP12-rapamycin binding) domain [57]. Rapamycin-induced dimerization of FKBP and FRB lead to the formation of an active PI-PLC enzyme at specific organelles, triggering the conversion of PI to diacylglycerol (DAG). The abundance of PI at different organelles can subsequently be estimated by measuring the local generation of DAG using a DAG-specific biosensor [57].

### Phospholipid biosensors based on dimerization-dependent fluorescent proteins (ddFPs)

An alternative approach is to use ddFP. In this approach, one of the ddFP monomers (copy A) contains a quenched chromophore and produces bright fluorescence upon binding with the other nonfluorescent monomer (copy B) [67, 105, 106] (Fig. 1d). The initial ddFP design was based on the dimeric red fluorescent protein dTomato [105].

Ratiometric biosensors have also been developed, which utilize the reversible exchange of copy B of green and red ddFPs, enabling qualitative imaging of various signaling activities [67]. For instance, the PH domain of PLCδ was fused to copy A of the red fluorescent version of ddFP and copy B, respectively. Dimerization of copy A and copy B resulted in red fluorescence at the plasma membrane. Upon carbachol stimulation and subsequent PtdIns(4,5)P_2_ hydrolysis, copy A and copy B dissociated from the plasma membrane. The released copy B could then bind to copy A of the green fluorescent version of ddFP in the cytoplasm, leading to an increase in the green-to-red ratio of the entire cell [67]. The ddFP method offers the advantage of lower background and enables the quantification of local lipid generation or fluctuation with higher sensitivity. Furthermore, by targeting the nonfluorescent copy B to specific membrane structures, such as the plasma membrane or endosomes using specific targeting sequences, the lipid-binding domain fused to copy A can be used as an organelle-specific lipid biosensor. However, ddFPs produce low levels of fluorescence, posing a challenge in the detection of phospholipid species with low abundance.

### Phospholipid biosensors based on FRET

FRET involves the non-radiative energy transfer from an excited donor fluorophore to a suitable ground-state acceptor fluorophore within nanoscale proximity (typically 1–10 nm) through dipole-dipole coulombic interactions [107–109]. FRET has been widely used to study the spatial and temporal dynamics of molecule conformational changes, molecule interactions, and signaling transduction at the whole-cell or single-molecule level in live cells [107, 109]. Based on whether the donor and acceptor fluorophores are fused on the same biomolecule, FRET biosensors, including lipid sensors, are classified as intramolecular or intermolecular types [108–110].

Compared to translocation-based lipid sensors, FRET lipid sensors offer the advantage of measuring lipid concentrations or turnover at small subcellular membrane regions, such as lamellipodial and filopodial protrusions and neurites [68]. However, implementing FRET-based sensors is technically more challenging as it has specific requirements for hardware and data analysis [107]. The number of available FRET-based lipid biosensors is still limited. With the advancement of novel donor-acceptor pairs and the implementation of single-molecule FRET in live cells [107, 111, 112], the sensitivity and performance of currently used sensors can be further improved.

The most straightforward intermolecular FRET lipid sensor can be created by fusing the lipid-binding domain with two spectrally compatible fluorescent proteins (Fig. 1e). For example, the PH domain of PLCδ1 was fused with a cyan fluorescent protein (CFP, as the donor) and yellow fluorescent protein (YFP, as the acceptor) to monitor PtdIns(4,5)P_2_ metabolism in the plasma membrane [68]. FRET occurred between PH(PLCδ1)-CFP and PH(PLCδ1)-YFP in resting cells. PtdIns(4,5)P_2_ hydrolysis led to the dissociation of PH(PLCδ1)-CFP and PH(PLCδ1)-YFP from the plasma membrane, thus resulting in cessation of FRET [68]. To achieve location-specific FRET signal detection, the donor component (a spectrally compatible fluorescent protein or a lipid-binding domain fused to a fluorescent protein) can be anchored to a specific subcellular membrane structure [30]. The FRET sensor pair can be expressed from two separate plasmids or a single vector containing self-cleaving viral 2A-peptide sequence [113].

Intramolecular FRET sensors are based on conformational changes induced by the binding of specific lipids to the designated lipid-binding domain (Fig. 1f). These sensors are typically fused with a specific membrane localization sequence to direct the sensor to the plasma membrane or intracellular membranes [69]. To examine the spatial-temporal dynamics of PtdIns(3,4,5)P_3_ in live cells, the PH domain from GRP1 was fused with CFP, YFP, and a membrane localization sequence at the C terminus through rigid linkers [69]. The binding of the PH domain to PtdIns(3,4,5)P_3_ during PI3K activation induced a conformational change in the sensor, leading to an altered intramolecular FRET from CFP to YFP [69]. A similar design has been employed to create intramolecular FRET sensors for PtdIns4P, PtdIns(4,5)P_2_, PtdIns(3,4)P_2_, and DAG, enabling the monitoring of dynamic turnover of different lipid species during growth factor stimulation and cell migration [114, 115].

### Phospholipid biosensors based on BRET

Like FRET, BRET involves the non-radiative transfer of energy from a bioluminescent donor molecule to an acceptor fluorophore. In BRET, the energy emitted from the bioluminescent enzyme donor (luciferase) upon substrate oxidation excites the acceptor fluorophore within nanoscale proximity (< 10 nm) [116]. Unlike FRET, BRET does not require an external light source for illumination [116]. BRET-based lipid biosensors are designed similarly to FRET sensors, where a specific membrane-targeting sequence is fused to the acceptor and a specific lipid-binding domain is fused to luciferase, which thus enables the measurement of lipid changes in specific cellular membranes [70] (Fig. 1g). For instance, the acceptor Venus was targeted to the plasma membrane using the plasma membrane-targeting sequence derived from Lck or c-Src [70, 71]. The donor *Renilla* luciferase was linked to lipid-binding domains such as tandem P4M domains for PtdIns4P, the PH domain of PLCδ1 for PtdIns(4,5)P_2_, the PH domain of Btk or GRP1 for PtdIns(3,4,5)P_3_, the tandem PH domains of TAPP1 for PtdIns(3,4)P_2_, and Lact-C2 for PS [70–72]. To express the acceptor and donor in a constant ratio (ideally 1:1), the donor and acceptor fusion proteins can be expressed from a single plasmid separated by the viral *Thosea asigna* virus (T2A) peptide [70–72]. The rapid change in lipid levels at the plasma membrane induced the membrane binding or dissociation of the lipid-binding domain, which effectively increased or reduced the BRET signal. Another approach for BRET sensor design is based on split luciferase complementation [73]. In this method, the N-terminal fragment of luciferase was anchored to the plasma membrane using a membrane-targeting motif, while the C-terminal fragment was fused with the PtdIns(3,4,5)P_3_-binding domain [73]. The binding of PtdIns(3,4,5)P_3_ by the PH domain brought the N-terminal luciferase fragment to the plasma membrane, leading to the complementation of the luciferase fragments and subsequent bioluminescence generation [73].

As the bioluminescence signal emitted from a single cell is rather weak, BRET measurements are typically conducted on cell populations [70, 72]. BRET-based sensors have been utilized to examine the agonist-induced generation of PtdIns4P, PtdIns(4,5)P_2_, PtdIns(3,4)P_2_, and PtdIns(3,4,5)P_3_ at the plasma membrane, as well as the concentration changes of PS and PtdIns4P following the recruitment of lipid transport proteins to the plasma membrane of cells in 96-well plates [70–72]. BRET measurements provide the population average and do not require light excitation, allowing for high-throughput imaging of lipid dynamics in cell lines stably expressing BRET sensors. This feature facilitates high-throughput screening by plate readers [73, 117].

### Phospholipid biosensors generated by chemical labeling of lipid-binding domains

Lipid-binding domains can also be chemically conjugated with an organic fluorophore. Environment-sensitive solvatochromic fluorophores, such as 2-dimethylamino-6-acyl-naphthalene (DAN) and Nile Red, exhibit a significant fluorescence blueshift upon binding to lipids in nonpolar environments in cellular membranes [74, 75] (Fig. 1h). A hybrid fluorescence sensor for PtdIns(4,5)P_2_, called DAN–eENTH, was developed by single-site chemical labeling of the mutated epsin1 ENTH (epsin N-terminal homology) domain with DAN [74]. The DAN–eENTH lipid sensor showed a blueshift in its emission spectrum, with the maximal emission wavelength shifting from 520 nm to 460 nm upon binding to large unilamellar vesicles containing an increased amount of PtdIns(4,5)P_2_ [74]. This sensor can be introduced into live mammalian cells through microinjection or the liposome-based protein delivery system. The PtdIns(4,5)P_2_ concentration in cellular membranes was quantified by a single-channel or ratiometric analysis at two wavelengths. Using this method, the average concentration of PtdIns(4,5)P_2_ in NIH3T3 cells was calculated to be 42 ± 6 nmol/m^2^ [74]. This chemical labeling method was also expanded to quantitative imaging of several other lipids, including PtdIns(3,4,5)P_3_, PtdIns(3,4)P_2_, PtdIns(4,5)P_2_, PtdIns(3,5)P_2_, PS, and cholesterol [42, 55, 75, 76, 118].

By incorporating two distinct solvatochromic fluorophores with minimal spectral overlap into either two different lipid-binding domains or the same lipid-binding domain (referred to as orthogonal sensors), it became possible to simultaneously image and quantify either two different lipid species at cellular membranes or a single lipid species in both leaflets of the plasma membrane [76]. For instance, the PH domain of myosin X was engineered with the Nile Red derivative NR3 (NR3-eMyoXPH) for PtdIns(3,4,5)P_3_ and DAN-eENTH for PtdIns(4,5)P_2_, enabling simultaneous quantification of the distribution and conversion of PtdIns(3,4,5)P_3_ and PtdIns(4,5)P_2_ at the plasma membrane [76]. Similarly, an orthogonal PtdIns(3,4,5)P_3_ sensor (DAN-eMyoXPH) and a PtdIns(3,4)P_2_ sensor (PH domain of TAPP1 labeled with NR3) were developed to facilitate simultaneous quantitative imaging of PtdIns(3,4,5)P_3_ and PtdIns(3,4)P_2_ [42]. Using this method, the concentrations of PtdIns(3,4,5)P_3_ and PtdIns(3,4)P_2_ at the plasma membrane were estimated to be around 0.10 mol% and 0.033 mol%, respectively [42]. Notably, simultaneous imaging of PtdIns(3,4,5)P_3_ and PtdIns(3,4)P_2_ revealed that while PtdIns(3,4,5)P_3_ was exclusively detected at the plasma membrane, PtdIns(3,4)P_2_ was found in both the plasma membrane and early endosomes [42]. The elimination of the PtdIns(3,4)P_2_ signal at early endosomes upon blockage of clathrin-mediated endocytosis indicated that clathrin-mediated endocytosis directly delivered locally generated PtdIns(3,4)P_2_ to early endosomes [42]. The orthogonal sensors have also been used to quantify PS or cholesterol concentrations and to monitor dynamic changes in the inner and outer plasma membranes of live cells [76, 118].

Recently, new fluorophores with highly desirable spectral and chemical properties were identified from a small library of solvatochromic fluorophores, which has further expanded the number of solvatochromic fluorophores suitable for ratiometric sensing [75]. It is important to note that the accuracy of sensor quantification depends largely on the sensitivity and specificity of the selected lipid-binding domain. The conjugation of solvatochromic dyes may affect the binding properties of the lipid-binding domains [119]. Furthermore, this method requires subtle protein engineering, and not all conjugated lipid-binding domains can exhibit the desired solvatochromic spectral properties [76]. Nevertheless, compared to lipid-binding domains fused with fluorescent proteins, lipid-binding domains conjugated with solvatochromic fluorophores offer a new approach for quantitatively measuring the distribution and concentration of different lipid species in live cells.

In this section, we have provided an overview of the design principles, applications, advantages, and limitations of different strategies employed in phospholipid biosensor design (Table 1). Given that each method possesses its own strengths and weaknesses, it is crucial to select the most suitable approach based on the specific biological questions.

## Validation of phospholipid biosensors

Validating phospholipid biosensors necessitates evaluating their specificity, selectivity, and sensitivity both *in vitro* and within cellular contexts, focusing on their reaction to alterations in specific lipid quantities or localizations [46, 62]. This can be accomplished by modulating lipid metabolism via genetic or pharmacological means or through inducible acute interventions [30, 40]. Genetic approaches, such as gene knockdown, knockout, or overexpression, offer effective strategies for altering enzyme activities linked to lipid metabolism. However, the complex feedback networks in cells often lead to redundancy and compensatory mechanisms that challenge the interpretation of long-term genetic interventions. To circumvent these issues, rapidly (milliseconds to minutes) inducible manipulation techniques have been developed, including electrogenetic, chemical-genetic, and optogenetic methods [30].

Chemical-genetic manipulation often involves rapamycin-induced dimerization of FKBP12 and FRB domains within the mammalian target of rapamycin (mTOR) pathway [120, 121]. Optogenetic strategies typically use light to induce dimerization of protein pairs, such as CRY2 (cryptochrome 2)-CIBN (the N-terminal portion of cryptochrome-interacting basic-helix-loop-helix 1 (CIB1)) [80, 122]. These methods allow for the precise recruitment of enzymatic domains to specific membranes, enabling acute modulation of phospholipid levels at targeted locations [30]. This acute modulation acts as a rigorous test for a biosensor’s dependency, selectivity, and sensitivity. Additionally, to test whether a phospholipid is sufficient for biosensor binding, targeted ectopic synthesis of the lipid is employed [62]. For instance, rapamycin-triggered recruitment of a PtdIns4P phosphatase to the plasma membrane rapidly decreases PtdIns4P levels, dislodging the PtdIns4P sensor EGFP-P4M from the membrane [34]. Conversely, ectopic production of PtdIns4P, through overexpression of specific enzymes, attracts EGFP-P4M to new membrane structures [34]. These experiments demonstrate the biosensor’s precise response to PtdIns4P levels, affirming that PtdIns4P’s presence is both necessary and sufficient for sensor recruitment [34].

## High-resolution imaging of phospholipids in live cells

Typically, lipid sensors, especially translocation-based sensors, are visualized by wide-field or confocal microscopy, allowing the characterization of lipid distribution and turnover at cellular or subcellular levels. Various super-resolution imaging techniques have emerged, which can provide new insights into the precise subcellular, organelle, or sub-organelle distribution of different phospholipids in live cells. However, lipids diffuse rapidly in cellular membranes: the lateral diffusion coefficient of lipid-binding domains typically ranges from 0.1 μm^2^/s to 1 μm^2^/s [1, 123–125]. The dissociation time of lipid-binding domains from the cell membrane can vary between 2 s and 7 s [123]. Moreover, different phospholipid species exhibit specific distribution and dynamic turnover at the membranes of various intracellular organelles. Among different imaging modalities, single-molecule imaging by TIRF microscopy is superior for analyzing the diffusion or interaction of individual lipid molecules at the plasma membrane. Advancements in light-sheet microscopy, especially lattice light-sheet microscopy, have enabled the tracking of molecule dynamics in three dimensions inside the live cells [126–128]. The high imaging sensitivity of TIRF microscopy and lattice light-sheet microscopy also helps to minimize any inhibitory effects by reducing the required cellular expression levels of lipid sensors. As conventional and super-resolution imaging methods are commonly used in biological labs and extensively discussed in other comprehensive reviews [29, 46], this review will briefly discuss the utilization of single-molecule imaging and lattice light-sheet three-dimensional (3D) imaging tools in dissecting the subcellular dynamics of lipids.

### Single-molecule kinetics of phospholipids in live cells

**Figure 3:**
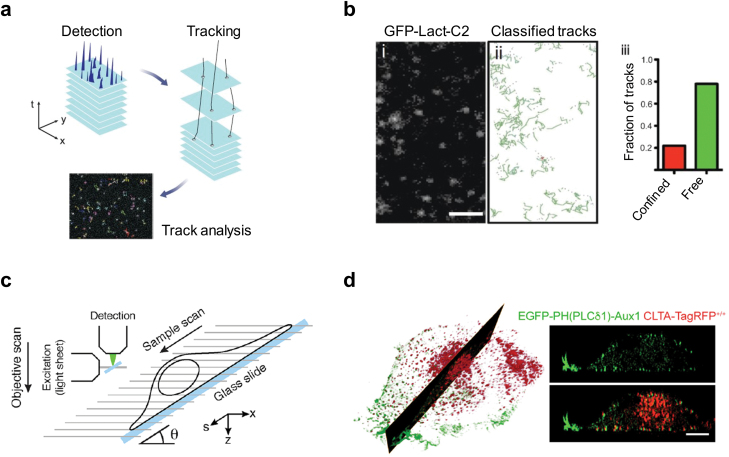
Single-molecule and 3D imaging of phospholipids in live cells. (a) Schematic overview of single-molecule imaging and analysis [129]. (b) TIRF imaging of HeLa cells expressing PS sensor GFP-Lact-C2. Shown are the representative image, classified tracks, and track classification analysis [130]. Scale bar, 2 μm. (c) Schematic representation of the experimental setup of lattice light-sheet microscopy [127]. (d) Lattice light-sheet microscopy imaging of genome-edited SUM159 cells expressing CLTA-TagRFP^+/+^ and the clathrin-coated structure-specific PtdIns(4,5)P_2_ sensor (EGFP–PH(PLCδ1)-Aux1). The 3D rendering and optical sectioning reveal the exclusive distribution of the sensor in clathrin-coated structures at the plasma membrane. The EGFP channel in the lower right panel was laterally shifted by six pixels [65]. Scale bar, 5 μm. Images reproduced with permission from [65, 127, 129, 130].

Single-molecule imaging offers quantitative information on the subcellular dynamic localization and kinetics of individual biomolecules in live cells with high spatial and temporal resolution [129] (Fig. 3a). Advancements in imaging modalities, fluorescent proteins and dyes, labeling strategies, and imaging analysis methods have made single-molecule imaging a vital tool for deciphering the kinetics of individual biomolecules and thus their collective behaviors, which are essential for understanding complex and dynamic biological processes like signal transduction, membrane trafficking, and gene regulation [131, 132]. TIRF microscopy is superior for assessing bulk lipid changes at the plasma membrane. It is the most widely used tool for quantifying the dynamic lateral diffusion or interaction of single lipid molecules at the single-molecule level at cellular membranes.

The evanescent field generated during total internal reflection decays exponentially with distance, significantly increasing the signal-to-background ratio and reducing the imaging background by selectively illuminating the sample within 100–200 nm of the cover glass [133]. Thus, TIRF microscopy is ideal for imaging and tracking biomolecules at or near the plasma membrane. The illumination depth of TIRF microscopy can be further extended while still maintaining single-molecule sensitivity using highly inclined and laminated optical sheet microscopy, which employs a highly oblique angle to create a thin optical sheet just above the optical substrate [131, 134]. To perform single-molecule studies in live cells using TIRF microscopy, it is crucial to keep the fluorescent spots at relatively low densities (1–3 molecules/μm^2^) to achieve accurate single-molecule detection within a diffraction-limited spot [135–137]. Several strategies have been utilized to achieve the labeling and tracking of single lipid molecules. The most straightforward approach is to express a conventional fluorescent protein (e.g. EGFP or mCherry) tagged with a lipid-binding domain at very low levels to capture well-separated single molecules [138]. This method has been applied to visualize PtdIns(3,4,5)P_3_ dynamics in living *Dictyostelium discoideum* cells. The sensor was made by fusing GFP with CRAC (cytosolic regulator of adenylyl cyclase), a PH domain-containing protein expressed in *Dictyostelium discoideum* cells [139]. A similar approach has been employed to quantify the diffusion dynamics of PS using EGFP-tagged Lact-C2 or TopFluor-PS (1-palmitoyl-2-(dipyrrometheneboron difluoride) undecanoyl-*sn*-glycero-3-phospho-l-serine, a synthetic fluorescent PS analog) in live HeLa cells [130] (Fig. 3b). However, the low signal-to-noise ratio and photobleaching of conventional fluorescent proteins hinder fast and long-term tracking of single lipid molecules. To overcome this limitation, single lipids in cells expressing lipid-binding domains fused with the self-labeling enzyme tag can be stochastically labeled with organic dyes [137]. Janelia Fluor dyes, with superior photostability and brightness, enable long-term single-molecule tracking with a high signal-to-noise ratio at various cellular membranes [94, 140]. Additionally, purified lipid-binding domains labeled with dyes can be injected into cells to achieve single-molecule labeling and imaging [42, 130].

Single-molecule imaging of phospholipids can be achieved by single-particle tracking photoactivated localization microscopy (sptPALM) with photoactivatable fluorescent proteins [141]. In each image frame, a small subset of photoactivatable fluorescent proteins are stochastically switched on by appropriate photoactivation light, enabling the precise determination of the locations of individual fluorophores above the resolution limit [142]. The sptPALM method has been utilized to characterize the diffusion kinetics of various lipid-binding domains tagged with the photoactivatable mCherry (PAmCherry)—such as cPHx3 of TAPP1 for PtdIns(3,4)P_2_, PH(PLCδ1) or Tubby_c_ for PtdIns(4,5)P_2_, P4M(SidM) and P4C(SidC) for PtdIns4P, and Lact-C2 for PS—at the plasma membrane of HeLa cells [44, 125]. Analysis of the reconstructed single-molecule trajectories revealed that these PAmCherry-tagged lipid-binding domains diffuse rapidly (with a diffusion coefficient of approximately 0.3 μm^2^/s) and freely (largely exhibiting Brownian motion) in the inner plasma membrane [125]. Interestingly, the lateral diffusion of PtdIns(4,5)P_2_ appears to be unaffected by membrane structures such as ER–plasma membrane contact sites [125]. Among several tested actin cytoskeletal elements, only spectrin and septin cytoskeletons affect PtdIns(4,5)P_2_ diffusion [125]. Thus, single-molecule imaging and analysis have aided in identifying the kinetics and mechanism of action of membrane lipids that are not observable using conventional imaging methods.

### 3D dynamics of phospholipids with lattice light-sheet microscopy

Light-sheet microscopy is an emerging technique for imaging cells and tissues in 3D. By illuminating the samples with a focused thin sheet of light from the side and detecting the emitted fluorescence with an orthogonal detection objective, light-sheet microscopy enables volumetric imaging with high temporal and spatial resolution. Among various light-sheet approaches, lattice light-sheet microscopy is particularly suitable for noninvasive 3D imaging and tracking of molecules and organelles in live cells [126–128]. The thin and nondiffracting two-dimensional (2D) light sheet generated by a 2D optical lattice uniformly spreads the excitation power while reducing light sheet thickness, which substantially minimizes photobleaching and phototoxicity while maintaining high speed and spatial resolution during live-cell imaging [126–128] (Fig. 3c).

Lattice light-sheet microscopy, with its high speed and near single-molecule imaging sensitivity, opens a new era for studying molecule dynamics at various intracellular membranes. This imaging technique, combined with quantitative imaging analysis, has been used to track the 3D distribution and dynamics of clathrin-coated structure-specific PtdIns(4,5)P_2_ and PtdIns(3,4)P_2_ sensors during clathrin-mediated endocytosis [65]. The coincidence detection sensor for PtdIns(4,5)P_2_ accumulated in clathrin-coated pits as they formed and disappeared upon clathrin-coat disassembly. Volumetric imaging with lattice light-sheet microscopy revealed the absence of the clathrin-coated structure-specific PtdIns(4,5)P_2_ sensor in endosomal membranes or the trans-Golgi network [65] (Fig. 3d). These imaging results are consistent with the pivotal roles of PtdIns(4,5)P_2_ in regulating the assembly and maturation of clathrin-coated pits at the plasma membrane [80, 143, 144] and the absence of PtdIns(4,5)P_2_ in clathrin-coated structures in endosomal membranes or the trans-Golgi network [4, 6]. The clathrin-coated structure-specific PtdIns(3,4)P_2_ sensor, generated using the tandem PtdIns(3,4)P_2_-binding PH domain of TAPP1, did not colocalize with clathrin-coated pits but appeared in clathrin-coated vesicles after scission [65] (Fig. 2c). This observation aligns with the finding that PtdIns3P instead of PtdIns(3,4)P_2_ is necessary for SNX9 (sorting nexin 9) recruitment to clathrin-coated pits [145]. Since the coincidence detection senor was recruited exclusively during coat disassembly but not assembly, it is less likely that the sensor recruitment was mediated by the putative clathrin-binding sequence within the C-terminal part of the PH domain (cPH) of TAPP1. Indeed, introduction of a point mutation (R212L) within the cPH of TAPP1 disrupted the binding of the TAPP1 domain to PtdIns(3,4)P_2_ [63, 146], resulting in the elimination of the recruitment of the PtdIns(3,4)P_2_-specific coincidence detector to coated vesicles [65]. These findings confirmed that the recruitment of the PtdIns(3,4)P_2_-specific coincidence detector to coated vesicles is dependent on the presence of PtdIns(3,4)P_2_. Importantly, it should be noted that attempting to interfere with the PtdIns(3,4)P_2_ level on budded vesicles by depleting PtdIns(3,4)P_2_ at the plasma membrane is not effective, as the endocytic vesicles are released and are no longer connected to the plasma membrane. With this endocytic vesicle-specific PtdIns(3,4)P_2_ sensor, the 3D dynamics of PtdIns(3,4)P_2_ and Ras-related protein in brain 5 (Rab5) in nascent endocytic vesicles were imaged and tracked using lattice light-sheet microscopy. By analyzing hundreds of uncoated vesicles originating from both the bottom and top surfaces of cells, it was found that the gradual increase in the EGFP-Rab5c signal overlaps the steady decrease of the PtdIns(3,4)P_2_ sensor signal. The quantitative imaging data provided further evidence supporting the exclusive presence of PtdIns(3,4)P_2_ in uncoating and uncoated vesicles, and demonstrated that uncoated endocytic vesicles recruit Rab5 before they fuse with a Rab5-positive early endosome [65].

Macropinocytosis is a clathrin-independent, nonselective endocytic pathway. Macropinosomes are formed from actin-enriched ruffles on the plasma membrane [147]. Similar to clathrin-mediated endocytosis, the formation of macropinosomes also involves the dynamic conversion of different phosphoinositide species [148]. Lattice light-sheet microscopy was employed for 3D imaging of the membrane structure, membrane dynamics, and coordinated PI3K activity during macropinosome formation [149]. Volumetric time-lapse imaging of the PI3K products PtdIns(3,4,5)P_3_ and PtdIns(3,4)P_2_ (labeled by the mScarlet-I-tagged PH domain of Akt) and the mNeonGreen-tagged plasma membrane marker revealed the recruitment dynamics of the mScarlet-I-tagged PH domain of Akt through the entire macropinosome formation process [149]. This study also uncovered that amplification of local PI3K activity within ruffles is required for the sealing of ruffles during macropinosome formation [149].

## Perspective

There are more than a thousand different lipid species in mammalian cells. However, a dynamic map of lipid distribution and kinetics in cells is still missing. From the perspective of lipid biosensor design, several emerging technologies are expected to provide new insights and expedite this process. Most currently used lipid-binding protein domains are derived from naturally occurring proteins, while high-avidity biosensors are only available for a limited number of lipid species. With the rapid development of *de novo* protein design, it is possible to create novel protein biosensors with high specificity and sensitivity [150]. The powerful *de novo* protein design tool can, therefore, be employed to create novel lipid-binding domains or modules specific to a wider range of lipids. Other technologies such as genome editing and machine learning have been applied in the design of biosensors for monitoring compartmentalized or multiplexed protein-mediated signaling transduction [151, 152]. These techniques can also be implemented in the design of novel lipid-binding sensors. It was recently reported that a nucleic acid RNA aptamer, identified from a screen, is specifically bound to PtdIns3P [153]. Aptamers are short single-stranded DNA or RNA molecules that can form highly ordered 3D structures and can recognize and interact with biomolecules with high affinity and specificity [154]. Thus, screening for aptamers specific to different lipids might offer a complementary approach for future lipid biosensor development.

Given that lipids diffuse rapidly and their turnover is transient, the ability to detect and track single lipids within live subcellular membranes with high spatial and temporal resolution is highly desired. Single-molecule imaging of dye-labeled genetically encoded lipid sensors using TIRF microscopy has become an ideal and straightforward tool for examining the diffusion kinetics of lipids at the plasma membrane of live cells. Several advanced imaging techniques, such as STED-FCS (fluorescence correlation spectroscopy in combination with super-resolution stimulated emission depletion microscopy), PALM, and lattice light-sheet microscopy, along with novel lipid biosensors, have significantly improved the imaging resolution and depth to the nanoscale and have enabled 3D imaging of phospholipids in live cells. The more recently developed MINFLUX (minimal photon fluxes) concept has achieved molecular precision (~1 nm) [155, 156]. Recent work has demonstrated that the MINFLUX concept can be implemented on a standard inverted fluorescence microscope to achieve 1–3 nm resolution in 3D in cells [156]. Additionally, lattice light-sheet microscopy combined with adaptive optics has paved the way for imaging the subcellular distribution and single-molecule kinetics of phospholipids in tissues [157]. The imaging ability and data analysis process can be further enhanced by various machine learning algorithms [158, 159].
